# DbcAMP regulates adipogenesis in sheep inguinal preadipocytes

**DOI:** 10.1186/s12944-017-0478-6

**Published:** 2017-05-19

**Authors:** Delin Kong, Jingjing Cui, Juncai Fu

**Affiliations:** 1 State Key Laboratory of Animal Nutrition, College of Animal Science and Technology, China Agricultural University, Beijing, 100193 People’s Republic of China; 2 China Agricultural University, Beijing, People’s Republic of China

**Keywords:** Preadipocyte, Proliferation, Differentiation, DbcAMP, Fatty acid, mRNA

## Abstract

**Background:**

The cyclic adenosine-monophosphate (cAMP) pathway is generally recognized as one of the essential pathways for the adipose conversion of rodent preadipocytes in vitro. However, divergent effects of cAMP on adipocyte differentiation have also been reported. Since there is very little data on non-rodent preadipose cells, the aim of the present work was to analyze the effects of one classic activator (dbcAMP) of the cAMP pathway on the proliferation and differentiation of sheep preadipocytes grown.

**Method:**

We retrospectively analyzed the regulation of dbcAMP on the proliferation and differentiation of sheep preadipocytes through observation on cell dynamic morphology, drawing on the growth curve, Oil Red O staining and induction of cell differentiation.

**Results:**

1) During first 5 days of treatment, at lower levels of dbcAMP (1 nmol/L to 1 × 10^4^ nmol/L), sheep cells were not increased, but at higher levels (1 × 10^5^ nmol/l to 1 × 10^6^ nmol/l), they were significantly increased (*P <* 0.05); 2) dbcAMP had the trendency to promote cell differentition, but it was not significant (*P >* 0.05); 3) treated for 4 days, dbcAMP at the levels of 1 nmol/L, 1 × 10^4^ nmol/L and 1 × 10^6^ nmol/L increased C20:0 abundance (*P <* 0.05), but other fatty acids had no significant changes; 4) treated for 4 days, expression of SCD mRNA had no significant change (*P >* 0.05), but expression of HSL mRNA increased at the level of 1 × 10^6^ nmol/L dbcAMP (*P <* 0.05).

**Conclusion:**

This study demonstrated that the mechanisms by which of the cAMP pathway affects on preadipocytes between sheep and rodent animals was different.

## Background

In recent years, as various cardiovascular diseases, obesity et al. become more and more universal, consumers have become more concerned about the possible health effects of excess fat in meat products. The amount of adipocyte growth (relative to muscle) is a major factor in determining the carcass merit and quality of the meat animal. Adipocytes mainly differentiate from preadipocytes in adipose tissue. So many studies about preadipocytes were conducted to explore the possible fat metabolism mechanisms and impact factors. Through the mechanisms and impact factors, fat metabolism can be regulated.

Cyclic adenosine monophosphate (cAMP) is a second messenger important in many biological processes. cAMP has been shown to mediate the hormonal regulation of lipid metabolism in fat cells from adipose tissue [1]. Fatty acid released from adipocytes is regulated by intracellular cAMP. Adipose tissue lipolysis is dependent on the intracellular concentration of cAMP, which is determined at the levels of both synthesis and degradation. Moreover, the cAMP analogue (dibutyryl cyclic AMP, dbcAMP) has the similar function of cAMP. Azarnia et al. found that addition of dibutyryl cyclic AMP and caffeine to already differentiated adipocytes resulted in loss of lipid. However, some studies had distinct results [2].

Stearoyl-CoA desaturase is a key enzyme involved in the synthesis of unsaturated fatty acids, as well as the regulation of this process. This enzyme catalyzes the rate-limiting step in the biosynthesis of monounsaturated fatty acids, Δ^9^-cis desaturation of fatty acids, from saturated fatty acids, in conjunction with the iron-containing, cytochrome b5, cytochrome P450, NADH (P)-cytochrome b5 reductase, and molecular oxygen. Although the insertion of a double bond occurs in a spectrum of methylene-interrupted fatty acyl-CoA substrates including trans-11 octadecenoic acid, the preferred substrates are palmitoyl-CoA and stearoyl-CoA, which are converted to palmitoleoyl-CoA and oleoyl-CoA, respectively. These monounsaturated fatty acids are used as substrates for the synthesis of triglycerides, wax esters, cholesteryl esters, and membrane phospholipids [3].

Hormone-sensitive lipase (HSL) is the key and the rate-limiting enzyme for hydrolyzing triacylglycerol to free fatty acid in fat tissues. And HSL is one of the most important factors for controlling the hydrolyzation of adipocyte tissues and fat accumulation in animals. The major physiological substrate for this enzyme is diacylglycerol. Triacylglycerol hydrolysis is stimulated by changes in the level of a variety of hormones (such as glucagon and epinephrine) that are elevated at times of energy need. These hormones bind to their respective receptors, stimulating a cascade of events that leads to the elevation of intracellular cAMP levels and results in the activation of protein kinase A (PKA). After adipocyte triacylglycerol lipase (ATGL) hydrolyzes triacylglycerols to generate diacylglycerols, the PKA-dependent phosphorylation of HSL, as well as the accessory protein perilipin, results in the stimulated hydrolysis of diacylglycerols, thereby generating monoacylglycerol and free fatty acids [4].

## Methods

### Cell isolation and culture conditions

One 3-month-old Dorper sheep which come from Beijing Aoxin Animal Husbandry Teaching and Research Base which belongs to China Agricultural University was killed by breaking its neck. Sheep inguinal adipose tissue was aseptically isolated, and visible connective tissue was removed. Tissue was then finely minced and incubated with 0.1% Type I collagenase, 2% (*w*/*v*) type V BSA, 137 mmol/l NaCl, 2.69 mmol/l KCl, 8 mmol/l Na_2_HPO_4_, 1.5 mmol/l KH_2_PO_4_, 100 U/ml penicillin, and 5 mg/ml streptomycin (all from Sigma, St. Louis, MO). The digestion period was 1 h at 37 °C with constant agitation in a shaking water bath; in turn, this was filtered through 147 and 45 μm nylon cell filters (Filcon; DAKO, Copenhagen, Denmark), then centrifuged for 8 min at 2000 rpm to obtain a stromal vascular cell pellet containing adipose precursor cells. The pellet was washed two times with DMEM. The adipocyte stromal vascular cells were plated in proliferation medium containing 90% DMEM (Sigma, St. Louis, MO), 10% FBS (PAA Laboratories, Linz, Austria), 100 U/ml penicillin, and 5 mg/ml streptomycin (all from Sigma, St. Louis, MO) at a density of 1 × 10^4^ cells/cm^2^ in 6 cm cell culture dishes (Corning Inc., Corning, NY). After 48 h of incubation in a 37 °C humidified atmosphere containing 5% CO_2_, medium was removed and the dishes were washed with DMEM to remove unattached cells and cell debris, and then medium was removed every 2 days [5].

### Induction of cell differentiation

Cells reached confluence in proliferation medium (Day 0). Cells were washed two times with PBS, then cultured for another 2 days in MDI induction medium (proliferation medium supplemented with 1 μmol/l dexamethasone, 0.5 mmol/l isobutylmethylxanthine, and 10 mg/l insulin, all from Sigma, St. Louis, MO). After two days of MDI induction, cultures were exposed to Insulin medium (proliferation medium supplemented with10mg/l insulin) for another two days. Thereafter, cells were exposed to proliferation medium and medium was removed every two days. Full differentiation is usually achieved by Day 8.

### dbcAMP preparation

dbcAMP were purchased from Sigma, St. Louis, MO. 0 nmol/l, 1 nmol/l, 10 nmol/l, 1 × 10^2^ nmol/l, 1 × 10^3^ nmol/l, 1 × 10^4^ nmol/l, 1 × 10^5^ nmol/l and 1 × 10^6^ nmol/l dbcAMP were prepared with proliferation medium.

### Experimental design

The objective of experiment 1 was to evaluate the effect of dbcAMP on the proliferation of sheep preadipocytes. Cells were incubated in six 96-well cell culture plates (Corning Inc., Corning, NY), every well containing 2 × 10^4^ cells. There were 8 treatments: 0 nmol/l, 1 nmol/l, 10 nmol/l, 1 × 10^2^ nmol/l, 1 × 10^3^ nmol/l, 1 × 10^4^ nmol/l, 1 × 10^5^ nmol/l and 1 × 10^6^ nmol/l dbcAMP. Every treatment had 4 replicates, and one well was one replicate. Cells were incubated respectively with 0 nmol/l (as a control), 1 nmol/l, 10 nmol/l, 1 × 10^2^ nmol/l, 1 × 10^3^ nmol/l, 1 × 10^4^ nmol/l, 1 × 10^5^ nmol/l or 1 × 10^6^ nmol/l dbcAMP for 6 days. Every day one 96-well cell culture cluster was determined by MTT Cell Proliferation Assay.

The objective of experiment 2 was to evaluate the effect of dbcAMP on differentiation of sheep preadipocytes. The cells were incubated in six 96-well cell culture plates (Corning Inc., Corning, NY), every well containing 1 × 10^5^ cells. There were eight treatments: 0 nmol/l, 1 nmol/l, 10 nmol/l, 1 × 10^2^ nmol/l, 1 × 10^3^ nmol/l, 1 × 10^4^ nmol/l, 1 × 10^5^ nmol/l and 1 × 10^6^ nmol/l dbcAMP. Every treatment had 4 replicates, and one well was one replicate. At confluence, cultured preadipocytes were induced to differentiate in MDI induction media for 2 days and then in Insulin media for 2 days, and treated respectively with 0 nmol/l (as a control), 1 nmol/l, 10 nmol/l, 1 × 10^2^ nmol/l, 1 × 10^3^ nmol/l, 1 × 10^4^ nmol/l, 1 × 10^5^ nmol/l or 1 × 10^6^ nmol/l dbcAMP for 12 days. Every two days, Oil Red O Cell Differentiation Assay determined one 96-well cell culture cluster [5].

The objective of experiment 3 was to determine the dose response of dbcAMP on mRNA transcript expression of SCD and HSL genes of sheep preadipocytes. The cells were incubated in 6-well cell culture plates (Corning Inc., Corning, NY), every well containing 2.5 × 10^6^ cells. There were five treatments: 0 nmol/l, 1 nmol/l, 1 × 10^2^ nmol/l, 1 × 10^4^ nmol/l and 1 × 10^6^ nmol/l dbcAMP. Every treatment had 3 replicates, and one well was one replicate. At confluence, cultured preadipocytes were induced to differentiate in MDI induction media for 2 days and then in Insulin media for 2 days, and treated respectively with 0 nmol/l (as a control), 1 nmol/l, 1 × 10^2^ nmol/l, 1 × 10^4^ nmol/l or 1 × 10^6^ nmol/l dbcAMP for 4 days. Then cells were obtained to determine mRNA transcript expression of SCD and HSL genes of sheep preadipocytes by quantitative real-time PCR.

Experiment 4 was designed to determine the effect of dbcAMP on the abundance of various fatty acids of sheep adipocytes. Cells were incubated in fifteen 10 cm cell culture dishes (Corning Inc., Corning, NY), every dish containing 1.37 × 10^7^cells. They were randomly divided into five treatments: 0 nmol/l, 1 nmol/l, 1 × 10^2^ nmol/l, 1 × 10^4^ nmol/l and 1 × 10^6^ nmol/l dbcAMP. Every treatment had 3 replicates, and one dish was one replicate. At confluence, cultured preadipocytes were induced to differentiate in MDI induction media for 2 days and then in Insulin media for 2 days, and treated respectively with 0 nmol/l (as a control), 1 nmol/l, 1 × 10^2^ nmol/l, 1 × 10^4^ nmol/l or 1 × 10^6^ nmol/l dbcAMP for 4 days. Then cells were obtained to determine abundance of various fatty acids of sheep adipocytes by gas chromatography method.

### MTT cell proliferation Assay

The reduction of tetrazolium salts is now recognized as a safe, accurate alternative to radiometric testing. The yellow tetrazolium salt (MTT) is reduced in metabolically active cells to form insoluble purple formazan crystals, which are solubilized by the addition of a detergent. The color can then be quantified by spectrophotometric means. A linear relationship between cell number and absorbance is established, enabling accurate, straightforward quantification of changes in proliferation.

Cultures were removed from one plate. Gently the plate was rinsed with 100 μl sterile PBS. Then PBS was removed and 25 μl MTT working solution was added to each well. After cells were incubated for 4 h in a 37 °C humidified atmosphere containing 5% CO_2_, MTT working solution was removed and cells were immediately rinsed with room temp ultrapure water until the water rinsed off clear. After all water was removed fully, each well added with 100 μl DMSO and was shaked slightly for 20 min at room temperature to be sure that all purple formazan crystals were in the solution. The solution OD was measured at 540 nm.

### Oil red O cell differentiation Assay

Cultures were removed from one plate. Gently the plate was rinsed with 100 μl sterile PBS. Then PBS was removed and 140 μl 10% formalin was added to each well. After cells were incubated for 25 min in a 37 °C humidified atmosphere containing 5% CO_2_, the formalin was removed, were discarded according to your chemical waste disposal procedure, and cells were immediately rinsed with room temp ultrapure water until the water rinsed off clear. After all water was removed fully, each well added with 140 μl 100% isopropanol and was shaked slightly for 20 min at room temperature to be sure that all sediments were in the solution. The solution OD was measured at 540 nm.

### Total RNA isolation and reverse transcription

In experiment 1, total RNA was isolated from the cultured cells using the RT reverse transcription Kit (Fermentas) according to the manufacturer’s protocol. The extracted RNA was dissolved in RNA-free water and quantified using ultraviolet-clear microplates (Corning Inc., Corning, NY) at an optical density of 260 nm. An RNA aliquot was verified for its integrity by electrophoresis on a 1.5% agarose gel stained with ethidium bromide. Then, 1 μg of total RNA was reverse-transcribed in a 20 μl reaction mixture using random primer Oligo-dT18 (Sangon, Shanghai, China) and Moloney murine leukemia virus reverse transcriptase (Promega, Madison, WI). The RT products (cDNA) were stored at −20 °C until analysis of selected gene mRNA levels by quantitative real-time PCR.

### Quantitative real-time PCR

Quantitative real-time PCR was performed using DNA Engine Opticon-2 (MJ Research, Waltham, MA) and SYBR Green PCR Master Mix Kits (ABI, America). GAPDH was used as the reference gene. The primers of the selected genes are listed in Table 1. The PCR system consisted of 10 μl of SYBR Green PCR Master Mix, 2 μl of cDNA, 6 μl of double distilled water, and 2 μl of primer pairs (25 μmol/l forward and 25 μmol/l reverse) in a total volume of 20 μl. Forty cycles were performed, each cycle consisting of denaturation (94 °C, 15 s), annealing (54 °C, 30 s), and elongation (72 °C, 45 s) except for the first cycle in which denaturation was 95 °C for 15 min and the last cycle in which the elongation time was for 10 min. The number of cycles used for each gene was in the linear amplification range. All samples were measured in triplicate. The relative mRNA levels of target genes were determined using the relative standard curve method.

**Table 1:** Primer sequences used for quantitative real-time PCR

Genes	Oligo	Primer sequence	Predicted size (bp)	Gene bank accession
HSL	Forward Primer Reverse Primer	5′-TGCCCAAGACAGAGCCAATG-3′ 5′-CCCAAGTAAGAAGTTGACGGTTGA-3′	209	NM_001128154
SCD	Forward Primer Reverse Primer	5′-GCTACAAGAGTGGCTGAGTTT-3′ 5′-AAGGCAGAGTTGTTGGTTTC-3′	185	NM_001009254
GAPDH	Forward Primer Reverse Primer	5′-GCAAGTTCCACGGCACAG-3′ 5′-GGTTCACGCCCATCACAA-3′	249	AJ431207

### Analysis of various fatty acids

The whole of cell pellet (about 1.37 × 10^7^ cells) in a glass methylation tube was mixed with 1 ml of N-hexane and 1 ml of 14% BF_3_/MeOH reagent. After blanketed nitrogen, the mixture was heated at 100 °C for 1 h, cooled to room temperature and methyl esters extracted in the hexane phase following addition of 1 ml ultrapure H_2_O. The samples were centrifuged for 1 min, and then the upper hexane layer was removed. Fatty acid methyl esters were analyzed by gas chromatography method [6].

## Results

### MTT cell proliferation Assay

**Fig. 1:**
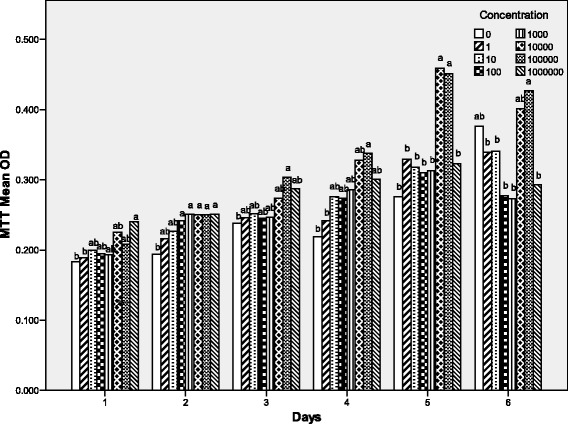
Effect of the dose of dbcAMP on the proliferation of sheep preadipocytes on Day1, Day2, Day3, Day4, Day5 and Day6. Sheep preadipocytes were grown in proliferation medium containing 0 nmol/l (as a control), 1 nmol/l, 10 nmol/l, 1 × 10^2^ nmol/l, 1 × 10^3^ nmol/l, 1 × 10^4^ nmol/l, 1 × 10^5^ nmol/l, or 1 × 10^6^ nmol/l dbcAMP for 6 days and the cell proliferation was determined by MTT Cell Proliferation Assay everyday of the 6 days. Statistical differences reported are among doses. Values are means ± SEM; *n* = 4. Values with different letters are significantly different (*P* < 0.05)

Fig. 1 showed that from Day1 to Day5, the cells at the low levels of dbcAMP (from 1 nmol/l to 1 × 10^4^ nmol/l) had no significant difference, but at the high levels, especially from 1 × 10^5^ nmol/l to 1 × 10^6^ nmol/l, they increased (*P <* 0.05). As culture time went on, the difference became more and more significant. However, on Day6, there was no significant difference.

### Oil red O cell differentiation Assay

**Fig. 2:**
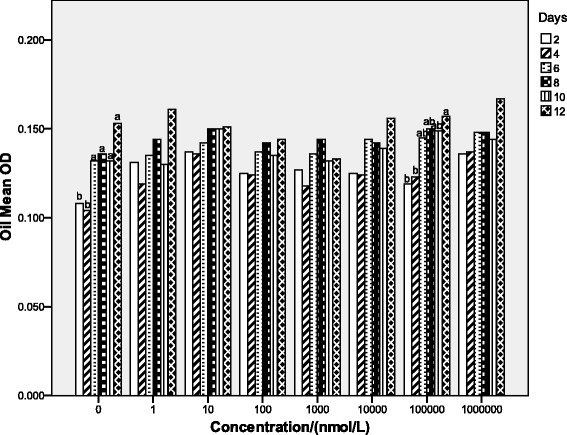
Effect of the dose of dbcAMP on the differentiation of sheep preadipocytes on Day2, Day4, Day6, Day8, Day10 and Day12. After cell differentiation induction, sheep preadipocytes were grown in proliferation medium containing 0 nmol/l (as a control), 1 nmol/l, 10 nmol/l, 1 × 10^2^ nmol/l, 1 × 10^3^ nmol/l, 1 × 10^4^ nmol/l, 1 × 10^5^ nmol/l, or 1 × 10^6^ nmol/l dbcAMP for 12 days and the cell differentiation was determined by Oil Red O Cell Differentiation Assay every two days of the 12 days. Statistical differences reported are among doses. Values are means ± SEM; *n* = 4. Values with different letters are significantly different (*P* < 0.05)

Fig. 2 showed that when cells were not treated by dbcAMP, as culture time went on, the differentiated cells increased significantly (*P* < 0.05). However, the varying doses of dbcAMP, except 1 × 10^5^ nmol/l, had no effect on the differentiation of sheep preadipocytes. At the level of 1 × 10^5^ nmol/l dbcAMP, as culture time went on, the differentiated cells increased significantly (*P* < 0.05). But on the same day, varied levels of dbcAMP had no significant effect on differentiation of sheep preadipocytes.

### Gene expression

**Fig. 3:**
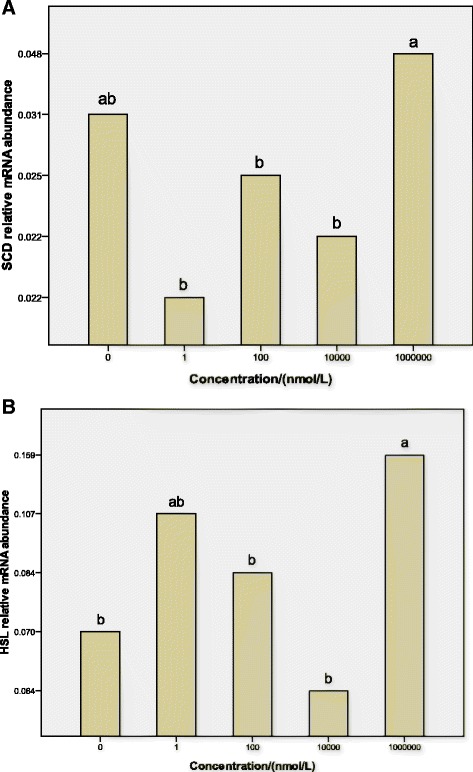
Effect of the dose of dbcAMP on the expression of SCD mRNA (**a**) and HSL mRNA (**b**) in sheep preadipocyte cultures. Sheep preadipocytes from inguinal adipose tissue were grown in proliferation medium containing 0 nmol/L (as a control), 1 nmol/l, 1 × 10^2^ nmol/l, 1 × 10^4^ nmol/l or 1 × 10^6^ nmol/l dbcAMP for 4 days after cell differentiation induction. Total RNA was harvested and used for first-strand cDNA synthesis. Quantitative real-time PCR analyses were performed to analyze the expression of SCD mRNA and HSL mRNA. The relative expression abundance of a given gene was calculated after normalization to GAPDH mRNA expression. Statistical differences reported are among doses. Values are means ± SEM; *n* = 3. Values with different letters are significantly different (*P <* 0.05)

Effects of the dose of dbcAMP on the expression of SCD mRNA and HSL mRNA of preadipocytes from sheep inguinal adipose tissue are shown in Fig. 3. After a 4-day treatment, no difference was found in the expression of SCD mRNA. And 1 × 10^6^ nmol/L dbcAMP increased significantly the expression of HSL mRNA (*P* < 0.05).

### Analysis of various fatty acids

**Fig. 4:**
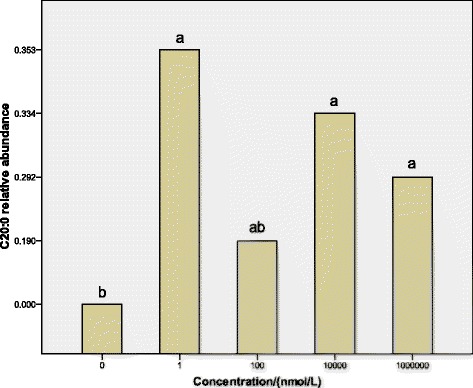
Effect of the dose of dbcAMP on the synthesis of various fatty acids in sheep preadipocyte cultures. Sheep preadipocytes were grown in proliferation medium containing 0 nmol/l (a vehicle control), 1 nmol/l, 1 × 10^2^ nmol/l, 1 × 10^4^ nmol/l or 1 × 10^6^ nmol/l dbcAMP for 4 days after cell differentiation induction. After various fatty acids of sheep preadipocytes were extracted, fatty acid methyl esters were analyzed by gas chromatography method. Only C20:0 at the different levels of dbcAMP had significant difference. Statistical differences reported are among doses. Values are means ± SEM; *n* = 3. Values with different letters are significantly different (*P* < 0.05)

The dose responses of dbcAMP on the synthesis of various fatty acids of sheep preadipocytes are showed in Fig. 4. After a 4-day treatment, the abundance of C20:0 was increased significantly by 1 nmol/l, 1 × 10^4^ nmol/l and 1 × 10^6^ nmol/l dbcAMP (*P <* 0.05), and at the level of 1 nmol/l the abundance of C20:0 was most. Other various fatty acids had no significant change.

The effect of dbcAMP on the synthesis of various fatty acids in sheep adipocytes are showed in Table 2.

**Table 2:** Effect of dbcAMP on the synthesis of various fatty acids in sheep adipocytes

Fatty acids	0 nmol/L	1 nmol/L	1 × 10^2^ nmol/L	1 × 10^4^ nmol/L	1 × 10^6^ nmol/L
C14:0	1.267 ± 0.600^a^	1.261 ± 0.110a	0.997 ± 0.149^a^	1.207 ± 0153^a^	1.470 ± 0.129^a^
C16:0	23.641 ± 3.026^a^	25.976 ± 0.707^a^	25.976 ± 0.707^a^	23.754 ± 3.247^a^	25.444 ± 0.901^a^
C16:1	1.559 ± 0.176^a^	1.585 ± 0.014^a^	1.246 ± 0.198^a^	1.419 ± 0.226^a^	1.495 ± 0.069^a^
C18:0	12.750 ± 0.468^a^	12.987 ± 0.378^a^	10.557 ± 1.702^a^	11.645 ± 1.731^a^	12.231 ± 0.345^a^
C18:1n7	6.919 ± 1.118^a^	7.726 ± 0.209^a^	6.330 ± 1.211a	7.162 ± 0.935^a^	7.797 ± 0.391^a^
C18:2n6	3.428 ± 0.534^a^	3.069 ± 0.146^a^	3.526 ± 0.536^a^	3.195 ± 0.666^a^	3.339 ± 0.428^a^
C20:0	0.000 ± 0.000^bB^	0.353 ± 0.047^aA^	0.286 ± 0.042^aA^	0.334 ± 0.063^aA^	0.292 ± 0.037^aA^
C20:1	4.095 ± 0.645^a^	4.723 ± 0.216^a^	3.707 ± 0.574^a^	4.436 ± 0.517^a^	3.973 ± 0.051^a^
C20:2	0.951 ± 0.070^a^	0.939 ± 0.082^a^	0.769 ± 0.128^a^	0.797 ± 0.101^a^	0.960 ± 0.122^a^
C20:4n6	7.887 ± 0.420^a^	7.840 ± 0.864^a^	7.026 ± 1.217^a^	7.201 ± 1.377^a^	8.391 ± 1.026^a^
C22:1	1.250 ± 0.048^a^	1.298 ± 0.038^a^	1.030 ± 0.102^a^	1.190 ± 0.155^a^	0.956 ± 0.036^a^
C22:4n6	0.350 ± 0.041^a^	0.365 ± 0.450^a^	0.517 ± 0.151^a^	0.470 ± 0.094^a^	0.314 ± 0.020^a^
C24:0	0.000 ± 0.000^bB^	0.000 ± 0.000^bB^	0.734 ± 0.077^aA^	0.000 ± 0.000^bB^	0.000 ± 0.000^bB^
C22:6n3	0.963 ± 0.033^a^	0.977 ± 0.063^a^	0.806 ± 0.129^a^	0.896 ± 0.152^a^	0.990 ± 0.091^a^
C24:1	1.509 ± 0.211^a^	1.628 ± 0.154^a^	1.411 ± 0.223a	1.501 ± 0.302^a^	1.514 ± 0.187^a^

There is no significant difference in the representation of the same lowercase letters (*P >* 0.05). The difference in marked lowercase letters is significant (*P <* 0.05). The difference between the different capital letters is very significant (*P <* 0.01)

## Discussion

To our knowledge, this study is the first to show the effect of cAMP pathway on the proliferation and differentiation of preadipocytes in sheep adipose tissue in vitro. Numerous in vitro studies have demonstrated the adipogenic effect of the cAMP pathway on rodent animals, but there is little data about non-rodent animals. This study aimed to study the adipogenic effect of cAMP pathway on sheep.

Numerous in vitro studies have demonstrated the adipogenic effect of the cAMP pathway, containing preadipocyte growth and differentiation. The role of the cAMP pathway in the regulation of mammalian cell proliferation and differentiation has been the subject of controversy. Divergent effects of the cAMP pathway on adipocyte differentiation have also been reported. The effects of the cAMP pathway on the differentiation of preadipocytes are somewhat controversial with stimulatory, inhibitory or neutral effects having been reported. These differences may be attributable to a number of reasons, including the use of different cell models and to the different conditions used to elicit differentiation [7]. Negative control was demonstrated in the 1970s, but evidence of positive control in other cell types has been neglected. One evidence which demonstrated such a control in the yeast *Saccharomyces cerevisiae* makes this concept acceptable [8]. Depending on the cell type, cAMP may act as a co-mitogen or promote differentiation. The cAMP pathway is generally recognized as one of the essential pathways for the adipose conversion of rodent preadipocytes in vitro. There is very little data on non-rodent preadipose cells. Addition of 8-bromo-cAMP was also found inefficient to stimulate porcine preadipocytes differentiation clearly. Similar culture conditions were adipogenic for the murine 3 T3-L1 preadipocytes but not for porcine preadipose cells. That work clearly highlighted the finding that porcine preadipocytes did not respond to classic activators of the cAMP pathway like rodent cells did. Addition of plasma-membrane-permeant cAMP analogs (8-bromocAMP, dibutyryl-cAMP) to cultured murine 3 T3-L1, murine Ob1771, or primary rat preadipocytes markedly enhanced lipid accumulation and also several markers of the adipose conversion process, including adipocyte P2 and stearoyl-CoA desaturase mRNAs, as well as glycerol 3-phosphate dehydrogenase (GPDH) and lipoprotein lipase (LPL) activities [9–11]. The positive effect of the cAMP pathway on adipocyte differentiation was further supported by the observation that two enhancers of the adenylyl cyclase pathway, adenosine A2 receptor and carbaprostacyclin (cPGI2), acting via G proteins, increased the GPDH activity in the murine Ob1771 preadipose cell line [12]. However, dibutyryl-cAMP(dbcAMP)in combination with theophylline (a phosphodiesterase inhibitor) was shown to prevent lipid accumulation in murine 3 T3-F442A cells [13]. High concentrations of intracellular cAMP potently inhibited preadipocytes 3 T3-F442A differentiation whereas low concentrations of intracellular cAMP, induced by a number of distinct agents, promoted differentiation [7]. 3 T3-L1 fibroblasts differentiation was restored by addition of dbcAMP. Christophe Boone, Francine Grégoire, and Claude Remacle (1999) found that when porcine preadipocytes were stimulated with agents increasing the intracellular cAMP level independently of membrane receptors: forskolin, 8-bromo-cAMP or MIX, these stimulators did not enhance the adipose conversion of porcine cells, whatever the culture medium (with or without serum) and the period of stimulation [13]. Both dbcAMP and forskolin negatively interfered with cellular proliferation of NIH 3 T3 cells as measured by [^3^H] thymidine incorporation [8]. In our study, in the beginning of cell growth, the high levels of dbcAMP, especially from 1 × 10^4^ nmol/l to 1 × 10^6^ nmol/l, promoted significantly the growth of sheep preadipocytes. dbcAMP had a very litte effect on the differentiation of sheep inguinal preadipocytes, but the effect was not significant. So these differences may be attributable to the use of different cell models.

Several studies have demonstrated adipocyte-specific genes that play important roles in the regulation of lipid metabolism and/or cell development, such as SCD, and HSL mRNA. It was reported that cAMP can activate HSL through cAMP-dependent protein kinase. David A. Casimir, et al. showed that SCD1 was induced during preadipocyte differentiation at two separate stages. There were three pieces of evidence demonstrating that the two inductions were distinct regulatory events. First, the early induction of SCD1 was driven by cAMP. cAMP did not affect SCD1 in fully differentiated adipocytes, the time of maximal late SCD1 expression. cAMP-elevating agents were used to differentiate preadipocytes and were removed from the adipogenic mixture after 48 h. Therefore, after 2 days, intracellular cAMP concentrations were returned to basal levels and could not act to stimulate SCD1 during the late induction. Second, chimeric CAT gene reporter constructs linked to the SCD1 cAMP-responsive region were activated by differentiating cells during the period of early induction but were not active during the later stage of differentiation. Third, prostaglandin F_2α_had been shown to inhibit the differentiation of 3 T3-L1 preadipocytes [14, 15]. Prostaglandin F_2α_-inhibited cells did not express late-induced SCD1 mRNA [14] but left the early induction intact [15]. These results demonstrated that the early induction caused by cAMP could be separated from the late effect. The transcriptional machinery responsible for the early SCD1 induction must be inactive during later differentiation. The early and late inductions were generated through separate mechanisms and probably played distinct roles in the differentiation of preadipocytes [11]. Our data clearly showed that any level of dbcAMP had no significant effect on the expression of SCD mRNA during late induction of sheep preadipocytes. This work clearly highlighted the finding that the early and late inductions were generated through separate mechanisms and probably played distinct roles in the differentiation of preadipocytes.

Hormone-sensitive lipase (HSL) catalyses the rate-limiting step in adipose tissue lipolysis and its activity is under acute hormonal and neuronal control. Noradrenalin, released from sympathetic nerve endings, and circulating adrenalin, corticotrophin and glucagon, all stimulate lipolysis by raising the intracellular concentration of cAMP. This leads to phosphorylation of HSL, causing activation of the enzyme and subsequent lipolysis. The major anti-lipolytic hormone is insulin which reduces phosphorylation of HSL, and acts, at least in part, by lowering the level of cAMP [16]. Jussi K. Huttunen and Daniel Steinberg showed that HSL purified approximately 100-fold from rat adipose tissue was activated 50 to 100% by incubation with cAMP, ATP-Mg^2+^ and a protein kinase preparation from rabbit muscle [17]. Studies in vivo and in vitro amply document the responsiveness of human adipose tissue to a variety of hormones, and indirect evidence has implicated cAMP in the process. Recently, HSL from rat adipose tissue has been partially purified and its activation has been shown to be effected via cAMP-dependent protein kinase [17]. Activation of HSL by cAMP in a cell-free system required Mg^2+^ and ATP. The effect of cAMP was greater on the lipolytic activity of a control homogenate than on an adrenalin-stimulated homogenate. That suggested that cAMP exerted a positive effect on the activation process rather than relieving MgATP^2−^ inhibition of the activated form of the HSL. One latter mechanism had been suggested for the action of cAMP on HSL. The demonstration of a cAMP-dependent protein kinase in adipose tissue might throw further light on the mechanism by which the cyclic nucleotide activated lipolysis [18]. Our data clearly showed that dbcAMP at the level of 1 × 10^6^ nmol/l had increased significantly the expression of HSL mRNA in sheep preadipocyte cultures with a 4 d treatment, which could increase hormone-sensitive lipase in sheep adipocytes. These results demonstrated that dbcAMP promoted sheep preadipocyte growth at the higher levels (1 × 10^4^ nmol/l to 1 × 10^6^ nmol/l) and HSL mRNA expression of sheep adipocytes at the higher level (1 × 10^6^ nmol/l), but had no effect on sheep preadipocyte differentiation and SCD mRNA expression of sheep adipocytes. Therefore the demonstration of the effect of the cAMP pathway on sheep preadipocytes may throw further light on the different mechanisms by which the cAMP activates lipolysis between rodent animals and non-rodent animals.

## Conclusions

dbcAMP had a very litte effect on the differentiation of sheep inguinal preadipocytes, but the effect was not significant.In conclusion,all these results demonstrated that the mechanisms by which of the cAMP pathway affects on preadipocytes between sheep and rodent animals was different.

## Abbreviations

ATGL: Adipocyte triacylglycerol lipase
cAMP: Cyclic adenosine-monophosphate
dbcAMP: Dibutyryl-cAMP
GPDH: Glycerol 3-phosphate dehydrogenase;
HSL: Hormone-sensitive lipase
LPL: Lipoprotein lipase
PKA: Protein kinase A

## References

[1] Butcher RW, Sutherland CE (1968). Effects of lipolytic and antilipolytic substances on adenosine 3′5′-monophosphate levels in isolated fat cells. J Biol Chem.

[2] Rahn LT. Down-regulation of cyclic-nucleotide phosphodiesterase 3B in 3T3-L1 adipocytes induced by tumour necrosis factor alpha and cAMP. Biochem. 2000;J.346:337–343. 10.1042/bj3460337 PMC1220858 10677351

[3] Ntambi JM (1992). Dietary regulation of Stearoyl-CoA Desaturase 1 Gene expression in mouse liver. J Biol Chem.

[4] Raben DM, Baldassare JJ (2005). A new lipase in regulating lipid mobilization: hormone-sensitive lipase is not alone. Trends Endocrinol Metab.

[5] Zhou X, Li DF, Yin JD, Ni JJ, Dong B, Zhang JX, Du M (2007). CLA differently regulates adipogenesis in stromal vascular cells from porcine subcutaneous adipose and skeletal muscle. J Lipid Res.

[6] Kang JX, Wang JD (2005). A simplified method for analysis of polyunsaturated fatty acids. BMC Biochem.

[7] Yarwood SJ, Kilgour E, Anderson NG (1998). Cyclic AMP potentiates growth hormone-dependent differentiation of 3T3-F442A preadipocytes: possible involvement of the transcription factor CREB. Mol Cell Endocrinol.

[8] Seternes, O. M., Sørensen, R., Johansen, B., and Moens, U. (1999). Activation of protein Kinase a by Dibutyryl cAMP treatment of NIH 3T3 cells inhibits proliferation but fails to induce ser-133 Phosphorylation and transcriptional activation of CREB.11, 211–219. 10.1016/s0898-6568(98)00069-2 10353696

[9] Björntorp P, Karlsson M, Pettersson P, Sypniewska G (1980). Differentiation and function of rat adipocyte precursor cells in primary culture. J Lipid Res.

[10] Brandes R, Arad R, Benvenisty N, Weil S, Bar-Tana J (1990). The induction of adipose conversion by bezafibrate in 3T3-L1 cells. Synergism with dibutyryl-cAMP. Biochim Biophys Acta.

[11] Casimir DA, Ntambi JM (1996). cAMP activates the expression of stearoyl-CoA desaturase gene 1 during early preadipocyte differentiation. J Biol Chem.

[12] Borglum JD, Vassaux G, Richelsen B, Gaillard D, Darimont C, Ailhaud G, Négrel R (1996). Changes in adenosine A1- and A2-receptor expression during adipose cell differentiation. Mol Cell Endocrinol.

[13] Boone C, Grégoire F, Remacle C (1999). Various stimulators of the cyclic AMP pathway fail to promote adipose conversion of porcine preadipocytes in primary culture. Differentiation.

[14] Casimir DA, Miller CW, Ntambi JM (1996). Preadipocyte differentiation blocked by prostaglandin stimulation of prostanoid FP2 receptor in murine 3T3-L1 cells. Differentiation.

[15] Miller CW, Casimir DA, Ntambi JM (1996). The mechanism of inhibition of 3T3-L1 preadipocyte differentiation by prostaglandin F2alpha. Endocrinology.

[16] Garton AJ, Campbell DG, Cohen P, Cohen P, Yeaman SJ (1988). Primary structure of the site on bovine hormone-sensitive lipase phosphorylated by cyclic AMP-dependent protein kinase. FEBS Lett.

[17] Huttunen JK, Steinberg D (1971). Activation and phosphorylation adipose tissue hormone-sensitive lipase by cyclic AMP-dependent protein kinase. Biochim Biophys Acta.

[18] Wade DR, Chalmers TM, Hales CN (1970). The effects of Mg^2+^, Ca^2+^, ATP and cyclic 3′,5′-AMP on the hormone-sensitive lipase of adipose tissue. Biochim Biophys Acta.

